# An O Island 172 Encoded RNA Helicase Regulates the Motility of *Escherichia coli* O157:H7

**DOI:** 10.1371/journal.pone.0064211

**Published:** 2013-06-13

**Authors:** Yanmei Xu, Xuefang Xu, Ruiting Lan, Yanwen Xiong, Changyun Ye, Zhihong Ren, Li Liu, Ailan Zhao, Long-Fei Wu, Jianguo Xu

**Affiliations:** 1 State Key Laboratory for Infectious Disease Prevention and Control (China CDC), Beijing, P R China; 2 National Institute of Communicable Diseases Control and Prevention and Control, Chinese Center for Disease Control and Prevention, Beijing, P R China; 3 Collaborative Innovation Center for Diagnosis and Treatment of Infectious Diseases, Hangzhou, China; 4 School of Biotechnology and Biomolecular Sciences, University of New South Wales, Sydney, New South Wales, Australia; 5 Network Information Center, Institute of Microbiology, Chinese Academy of Sciences, Beijing, P R China; 6 Laboratoire de Chimie Bactérienne, UPR9043, Université de la Méditerranée Aix-Marseille II, Institut de Microbiologie de la Méditerranée, CNRS, Marseille, France; U. S. Salinity Lab, United States of America

## Abstract

Enterohaemorrhagic *Escherichia coli* (EHEC) O157:H7 is a major cause of zoonotic food- and water-borne intestinal infections worldwide with clinical consequences ranging from mild diarrhoea to hemolytic uraemic syndrome. The genome of EHEC O157:H7 contains many regions of unique DNA that are referred to as O islands including the Shiga toxin prophages and pathogenicity islands encoding key virulence factors. However many of these O islands are of unknown function. In this study, genetic analysis was conducted on OI-172 which is a 44,434 bp genomic island with 27 open reading frames. Comparative genome analysis showed that O1-72 is a composite island with progressive gain of genes since O157:H7 evolved from its ancestral O55:H7. A partial OI-172 island was also found in 2 unrelated *E. coli* strains and 2 *Salmonella* strains. OI-172 encodes several putative helicases, one of which (Z5898) is a putative DEAH box RNA helicase. To investigate the function of Z5898, a deletion mutant (EDL933ΔZ5898) was constructed in the O157:H7 strain EDL933. Comparative proteomic analysis of the mutant with the wild-type EDL933 found that flagellin was down-regulated in the Z5898 mutant. Motility assay showed that EDL933ΔZ5898 migrated slower than the wild-type EDL933 and electron microscopy found no surface flagella. Quantitative reverse transcription PCR revealed that the *fliC* expression of EDL933ΔZ5898 was significantly lower while the expression of its upstream regulator gene, *fliA*, was not affected. Using a *fliA* and a *fliC* promoter – green fluorescent protein fusion contruct, Z5898 was found to affect only the *fliC* promoter activity. Therefore, Z5898 regulates the flagella based motility by exerting its effect on *fliC*. We conclude that OI-172 is a motility associated O island and hereby name it the MAO island.

## Introduction

Enterohaemorrhagic *Escherichia coli* (EHEC) O157:H7 is a major cause of zoonotic food- and water-borne infections worldwide. The genome of *E. coli* O157:H7 strain EDL933 contains 177 O-islands (OIs) in comparison to *E. coli* K12 MG1655 [1]. These islands encode 26% of the EDL933 genes (1,387/5,416) [1]. Several OIs are known to encode key virulence factors for O157:H7 including adhesins, a type III secretion system (T3SS) and its effectors, and the Shiga toxins (Stx1 and Stx2), while a number of O islands are involved in the regulation of virulence and environmental adaptation [2]–[16].

OI-148 contains the locus of enterocyte effacement (LEE) pathogenicity island and is required for full virulence of O157:H7 [2]–[5]. OI-122 carries the virulence gene *efa1*-*lifA* encoding the EHEC adherence/lymphocyte inhibitory factor, which is involved in the colonization of the intestinal mucosa and in the inhibition of the host immune response [6]–[8] and has been frequently found in Shiga toxin *E. coli* strains associated with severe human disease [9]. OI-15 contains the AidA15 adhesin gene, which plays a role in adherence [10], [11]. OI-48, consists of three functional gene clusters that encode urease, tellurite resistance, and adhesins Iha and AIDA-1, which may contribute to EHEC O157:H7 pathogenesis by promoting adherence of the pathogen to the host intestinal epithelium [12]. OI-1 encodes a putative fimbriae as well as a gene, *fmrA*, that represses flagellar synthesis and inhibits motility [13]. Eighteen of the OIs in EDL933 are prophages [1]. Apart from the Shiga toxin prophages which play an essential role in disease, prophage island CP-933P encodes a non-LEE-encoded T3SS effector, NleA [14]. Prophage island CP933-N (OI-50) represses the LEE-encoded T3SS [15]. Prophage island CP-933H encodes an AraC-like regulator, PatE, that activates the transcription of the *hdeAB*-*yhiD* cluster and other acid resistance operons, which greatly enhances the ability of O157:H7 to survive in different acidic environments [16].

In this study, we found OI-172 plays a role in regulating flagellar synthesis. The bacterial flagellum is a complex macromolecular structure driven by a motor which rotates a long curved filament extending from the cell envelop [17]. This filament is composed of a polymer of flagellin subunits encoded by the *fliC* gene [18], [19]. The synthesis and assembly of flagella is controlled by three classes of genes [18], [20]. Class I genes consist of one operon encoding FlhD and FlhC proteins which form a transcriptional complex that binds upstream of Class II promoters [21], [22]. Class II genes encode proteins for assembly of the basal apparatus and hook, as well as the class III regulator FliA. Class III genes encode proteins for the rest of the structure of the flagellum with the *fliC* gene encoding the major flagellin. The regulation of flagellar expression is controlled by many factors such as MatA [23], CRP [24], [25], Fur [26], [27], OmpR[27], H-NS [25], HdfR [28], LrhA[29], QseBC[30], RcsAB[31], DksA and ppGpp [32] and also by sRNAs [33].

OI-172 in EDL933 is a 44,434 bp genomic island with 27 open reading frames (ORFs) including two putative integrases (Z5878 and Z5890), one putative tansposase (Z5880), one putative resolvase (Z5885), several putative helicases including a DEAH box RNA helicase (Z5898), and 17 genes of unknown function. The putative DEAH box RNA helicase, Z5898, is of particular interest. The DEAH box [a motif named after its amino acid sequence (aspartate-glutamate-alanine-histidine) box motif] subfamily of RNA helicases is characterized by the presence of several conserved motifs [34]. These proteins are found virtually in all organisms and perform important roles from all aspects of RNA metabolism to transcriptional regulation [35]. DEAH box RNA helicases belong to the superfamily 2 of RNA helicases which is the largest and most diverse helicase superfamily [36] and are widely distributed in bacteria with up to 12 DEAH box RNA helicases per genome [37]. There are 5 well studied DEAD-box RNA helicases (DeaD/CsdA, RhlB, RhlE, SrmB and DbpA,) in *E. coli* that are involved in ribosome assembly and translation, and RNA degradation, which regulates gene expression at transcriptional and translational levels [38]. Here we report that the putative DEAH box RNA helicase, Z5898, encoded by OI-172 acts as a regulator of flagellum at *fliC* transcriptional level. OI-172 is thus a motility associated O (MAO) island.

## Materials and Methods

### Bacterial Strains, Plasmids and Bacterial culture

Bacterial strains and plasmids used in this study are listed in Table 1. Bacteria were grown in Luria-Bertani (LB, Miller) broth or agar. LB is suitable and widely used media for flagella expression in *E. coli*. However, the fluorescence background in LB is very high. We switched to MEM which has significantly reduced background. For the analysis of *fliA* and *fliC* promoter expression, MEM-HEPES (Sigma-Aldrich) supplemented with 250 nM Fe(NO_3_)_2_ and 0.2% glucose was used for culturing strains rather than LB as the background fluoresce was much lower when MEM was used. When required, L-arabinose and antibiotics were added to the media at the following concentrations: 0.2% L-arabinose, 100 µg ml^−1^ ampicillin, 50 µg ml^−1^chloramphenicol and 50 µg ml^−1^ kanamycin. We performed most of our motility related experiments at an OD of 0.6 at which density bacterial growth is in the log phase.

**Table 1:** Strains and plasmids used in this study.

Strains/plasmids	Description	Source
EDL933	ATCC strain	ATCC
EDL933Δ5898	Z5898 gene replace by kan gene from pRS551 in EDL933	This study
DH5α	Chemical competent cell	TaKaRa
pRS551	A 1696 bp *Pvu*II fragment containing the kan gene and promoter from Tn903 inserted between bla and Tl4 in pRS415	[54]
pKOBEG	A thermosensitive replicon that carries the λ phage *redγ*βα operon expressed under the control of the arabinose-inducible pBAD promoter	[55]
pBAD/*Myc*-His A	Arabinose inducible expression vector for His tagged fusion proteins	Invitrogen
pBADZ5898	A 6315 bp Z5898 fragment cloned into pBAD/*Myc*-His A between *Pst*I and *Ecor*I sites	This study
pAJR70	pACYC184 cut *BamH*I, egfp gene cloned into *BamH*I and *Bgl*II sites	[56]
pAJRfliA	*fliA* promoter cloned into pAJR70 between *BamH*I and *Kpn*I sites	This study

### Construction of Z5898 deletion mutant in EDL933

Replacement of Z5898 on the *E. coli* O157:H7 EDL933 chromosome by the Km^r^ gene was performed using one step method as described by Datsenko and Wanner [39]. The Km^r^ gene was amplified by PCR from plasmid pRS551 using primer pair P3 and P4 (Table 2). The gene deletion mutant (EDL933Δ5898) was confirmed by PCR and sequencing. The primer pairs P1 and P2, P5 and P6 (Table 2) were used to confirm gene deletion.

**Table 2:** Primers used in this study.

Primer name	Sequence (5′–3′)$	Source/Target
P1	gaggtgagggacgcaataac	Z5898
P2	ctctttgtggcgggtaaatg	Z5898
P3	caagagaagagcagttaggaccgtttaatggtcgacgcttcacgttgtgtctcaaaatct	[57]
P4	tgatgcgcaccgccccggtgggtaaggcgggcagtaaatccgtcccgtcaagtcagcgta	[57]
P5	ccagagagggaaaatacgca	Z5898
P6	gtgactaacgaccagaaacg	Z5898
5-Z5898F	aaaaa**ctgcag**catggacgttcagcaactgcattatgc	Z5898
3-Z5898R	aaaaa**gaattc**cttatccagcgataatataactcctaagg	Z5898
fliAF	cc**ggatcc**cctgattaactgagactgacg	*fliA*
fliAR	cc**ggtacc**gacataacgctgccacagc	*fliA*
fliCF	ccggatccccatgcgatttccttttatcat	*fliC*
fliCR	cc**ggtacc**gcagactggttcttgttgata	*fliC*
qRTfliCF	acaacgctggtagcgcagct	*fliC*
qRTfliCR	ggcagccgctttggtttcgc	*fliC*
qRTgapAF	ggttttctgagtagcggtagtagc	*gapA*
qRTgapAR	tatgactggtccgtctaaagacaa	*gapA*
qRTfliAF	ggataaacactcgctgtggcag	*fliA*
qRTfliAR	gaagttcatccagcatagcgcc	*fliA*

$ Bold sequences are restriction sites.

### Construction of plasmids for the complementation of Z5898 and the expression of *fliA* and *fliC*

The Z5898 complementation plasmid was constructed using PCR products amplified from EDL933 using primers Z5898F and Z5898R (Table 2) and cloned into pBAD/*Myc*-His A (Table 1). The resulted plasmid was named as pBADZ5898. *fliA* and *fliC* promoters were amplified from EDL933 using fliAF and fliAR; fliCF and fliCR and cloned into pAJR70 to create pAJRfliA and pAJRfliC. These two plasmids provided readout of *fliA* and *fliC* transcriptional activity and any transcriptional regulation acting on this promoter. *E. coli* strain DH5α was used as the intermediate host strain for cloning and all constructs were verified by sequencing.

### Cellular fractionation, 2D electrophoresis, and mass spectrometry

To prepare cellular fractions, bacteria were grown under identical conditions in 200 ml of LB medium and the cells were harvested by centrifugation at 17,000 *g* for 10 min. Spheroplasts were washed once and disrupted by sonication in 2 ml of 40 mM Tris-HCl (pH 7.6), in a Branson Sonifier 450 in the continuous mode and with an output setting of 2 for 30 s. The two-dimensional gel electrophoresis (2DE) was performed according to Amersham Biosciences' instructions. For mass spectrometry, protein samples were separated by 2DE and stained by Coomassie blue or silver staining and the specific protein spots were excised. After crushing and washing of the excised gel, the proteinaceous material was reduced with dithiothreitol and alkylated with iodoacetamide in 100 mM NH_4_HCO_3_. Proteolytic digestion by trypsin was then performed overnight at 37°C. The supernatant was collected and the salts were removed by flowing through an R2 Poros column. The sample was then analyzed by mass spectrometry. The protein was identified by using the ProFound software (http://www.matrixscience.com).

### Motility assays

Swimming motility was evaluated using soft agar plates. Agar (0.3%, Sigma) was added to 1% Bacto-Tryptone (BD; Becton Dickinson) broth containing 0.5% NaCl (Sigma-Aldrich). For plasmid-based complementation experiments, ampicillin and arabinose were added when appropriate and all plates were air dried overnight. Plates were stab inoculated with standardized overnight cultures (OD_600_ of 1.0) of *E. coli* EDL933 and its mutant using a sterile inoculating needle and incubated at 37°C for 16 h. All strains were tested in triplicate and each experiment was carried out on three separate occasions.

### Western blot analysis

To investigate the levels of H7 flagella expression in EDL933 and Z5898 mutant strains, bacteria were grown overnight in LB at 37°C and then cultured as 1∶100 dilution till to an OD_600_ of 0.6. Whole-cell lysates were resolved on a 12% sodium dodecyl sulfate-polyacrylamide gel. The gels were transferred onto nitrocellulose membranes (Millopore) for immunoblotting. The immobilized proteins were incubated with primary antibodies against H7 flagellin (Statens Serum Institut, Denmark) and detected by adding a secondary antibody with IRDye 800-labeled anti-rabbit IgG (Rockland, Gilbertsville, PA, U.S.).

### Transmission electron microscopy (TEM)

Wild-type EDL933 and its Z5898 deletion mutant were cultured in LB broth at 37°C till OD_600_ of about 0.6. Bacteria were dropped to parchment paper and adsorbed on 200-mesh copper TEM grids by laying the grids down on the parchment paper for 1 min. Bacteria on TEM grids were stained by submerging the grids for 30 s in 1% (wt/vol) phosphotungstic acid and then examined with a Philips Tecnai 12 transmission electron microscope at an operating voltage of 80 kV. Digital images of bacteria were captured with Gatan Digital Micrograph Imaging System by a Erlangshen CCD camera (Gantan).

### RNA extraction, cDNA synthesis and quantitative reverse transcription PCR

Overnight cultures of *E. coli* EDL933, its mutant and complemented strain were diluted 100-fold in LB broth and then grown to an OD _600_ of 0.6 with shaking. Total RNA was extracted using RNeasy Mini Kit (Qiagen) following the manufacturer's instructions. RNA was treated with DNase I (NEB). Expression of *fliC* and *fliA* was quantified by quantitative reverse transcription-PCR (qRT-PCR) analysis. Reverse transcription was performed using PrimeScript® RT reagent Kit (Perfect Real Time) (TaKaRa). qRT-PCR was carried out using SYBR® Premix Ex Taq^TM^ II (Perfect Real Time) (TaKaRa) using a Rotor-Gene Q thermal cycler (QIAGEN). Data was analyzed with Rotor-Gene Q Series Software, version 1.7 (QIAGEN). Data were normalized to the endogenous reference gene *gapA* and analyzed by the cycle threshold method (2^−ΔΔ*CT*^) ([40]. Three independent isolated cDNA samples were analyzed. Primers for amplifying *gapA*, *fliC* and *fliA* are detailed in Table 2 with qRTgapAF and qRTgapAR for *gapA*, qRTfliAF and qRTfliAR for *fliA*, qRTfliCF and qRTfliCR for *fliC*.

## Results

### Comparative analysis of genetic structure of the OI-172 of *E. coli* O157:H7

**Figure 1:**
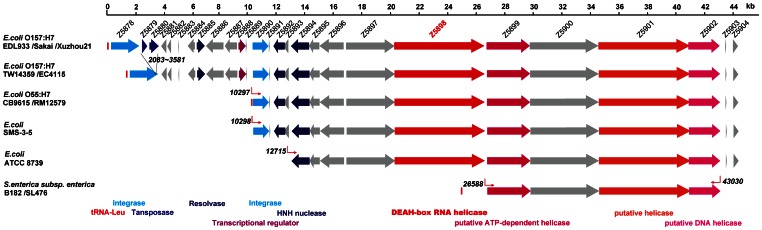
Architecture of OI-172 (MAO island) in *E. coli* O157:H7 and its homologs in other strains. The DNA sequence of the MAO island of *E. coli* O157:H7 strain EDL933 was used to search for homologous genes in the GenBank by BLASTn. The scale bar and the locus_tags as annotated in the EDL933 genome are indicated at the top row. Genes of unknown function are shown in grey. Other genes are coloured as shown at the bottom of the figure with putative functions in coloured text. The same color code was used if the genes have the same or similar function. The Genbank accession numbers of the complete genomes used are BA000007 (Sakai), CP001925 (Xuzhou21), CP001368 (TW14359), CP001164 (EC4115), CP001846 (CB9615), CP003109 (RM12579), CP000970 (SMS-3-5), CP000946 (ATCC 8739), CP003416 (B182) and CP001120 (SL476).

The OI-172 in EDL933 is a 44,434 bp genomic island with 27 open reading frames (ORFs) including two putative integrases (Z5878 and Z5890), one putative tansposase (Z5880), one putative resolvase (Z5885), 4 putative helicases (Z5898, Z5899, Z5901 and Z5902), and 17 genes of unknown function (Figure 1). The OI-172 is adjacent to a leu-tRNA locus with a G+C content of 47.3%. The OI-172 is present in the other 4 completely sequenced O157:H7 genomes, Sakai, Xuzhou21, TW14359, and EC4115 (Figure 1). In Sakai and Xuzhou21, all genes are present while 2 genes are absent in TW14359 and EC4115. Since TW14359 is known to be diverged earlier [41], it seems that the most recent common ancestor of EDL933, Sakai and Xuzhou21 gained an additional 2 genes (Z5879 and Z5881) after diverged from TW14359. The OI-172 is also partially present in the O55:H7 strain CB9615 with only the right hand side of the island present. The left hand side (ORF Z5878 to Z5889) of the OI-172 is likely to have been gained by O157:H7 after divergence from O55:H7 since O157:H7 is known to have derived from an O55:H7 strain [42], although it could have also been lost in O55:H7.

We performed BLAST searches against other *E. coli* genomes and other *enterobacteriaceae* genomes and found that two other *E. coli* strains unrelated to O157:H7-O55:H7 lineage, SMS-3-5, a multi-drug resistant environmental isolate [43] and ATCC 8739, a non-pathogenic laboratory strain, contain a partial OI-172 from Z5890 and Z5894 to the end of the OI-172 respectively. Four genes (Z5899 to Z5902) were also found in 2 *Salmonella enterica* strains as a contiguous block with high identity ranging from 82.54% to 86.21% at DNA level.

**Figure 2:**
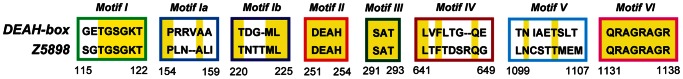
Schematic representation of conserved DEAH-box RNA helicase motifs in the Z5898 of *E. coli* O157:H7 EDL933. The DEAH-box conserved motifs are described by Cordin *et al*. [58]. The sequence and location of conserved motifs of the DEAH-box family aligned with Z5898 are shown. The yellow shadow show the perfect matches. The numbers below the boxes are amino acid positions of Z5898.

The four putative helicases encoded by the OI-172 shares very little similarity with each other and with other helicases on the chromosome, five of which contain DEAH boxes [44]. Z5898 have 1 DEAH box motif and other DEAH-box family conserved motifs as shown in Figure 2.

### Effect of Z5898 on protein expression

**Figure 3:**
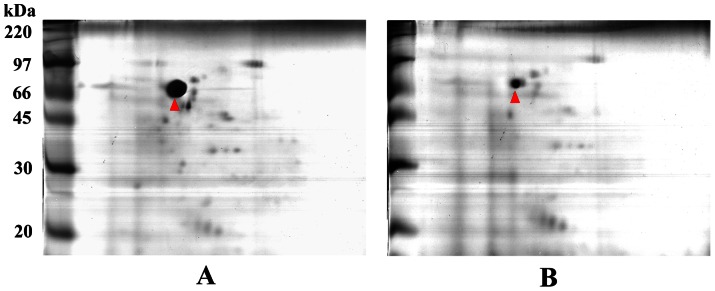
2-D gel electrophoresis patterns. 2-D gel electrophoresis patterns of proteins isolated from cells of *E. coli* O157:H7 EDL933 (A) and its isogenic mutant derivative EDL933ΔZ5898 (B). Rainbow marker was used as standard molecular weight. The sizes for each band were labelled. The spots indicated by arrowheads were flagellin identified by mass spectrometry.

Z5898 was selected for functional analysis since its putative function is a DEAH box RNA helicase and is only present in *E. coli*. It was deleted in EDL933 resulting in a mutant strain designated as EDL933ΔZ5898. A comparative proteomic analysis of whole cell extracts from the lysis of spheroplasts between wild-type EDL933 and EDL933ΔZ5898 mutant was conducted and found one spot (indicated by arrowheads in Figure 3) that was strongly differentially expressed. The spot was identified by mass spectrometry as a flagellin protein. There were other minor protein spots that are potentially differentially expressed and were not pursued further in this study.

### Z5898 regulates motility

**Figure 4:**
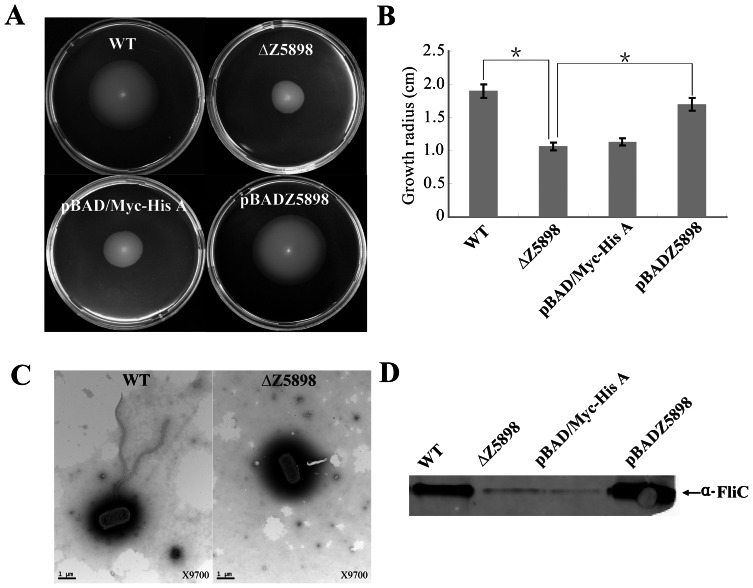
Effects of Z5898 on motility. (A, B) Representative images and growth radius of swimming motility for the wild-type EDL933, Z5898 deletion mutant (EDL933ΔZ5898), and complemented strain (EDL933ΔZ5898+pBADZ5898). pBAD/*Myc*-His A is an empty vector control. Error bar shows the standard deviation from three independent experiments. Differences were analyzed for significance by using T-test. Significant difference between two strains (P<0.01) are indicated by a * with a linked line. (C) Transmission electron micrographs of wild-type EDL933 and Z5898 mutant (scale bar, 1 μm). (D) Immunoblot analysis of FliC protein in the whole cell lysates prepared from wild-type EDL933, Z5898 deletion mutant (EDL933ΔZ5898), empty vector control strain (EDL933ΔZ5898+pBAD/*Myc*-His A) and complemented strain (EDL933ΔZ5898+pBADZ5898) grown in LB. Arrows indicate a reactive band corresponding to FliC detected with anti-H7 FliC antibodies.

Since flagellin expression was the main difference between EDL933 and EDL933ΔZ5898, motility difference was assessed. The radius of chemotactic ring was used as a measure of motility and was 1.90 cm and 1.07 cm for EDL933 and EDL933ΔZ5898 respectively (Figure 4A–B). The difference is statistically significant (t test, *P*<0.01). Thus the motility in the mutant was repressed in comparison to the wild-type EDL933. To determine whether the ΔZ5898 deletion can be complemented, we created pBADZ5898, a low copy number plasmid carrying Z5898 which was transformed into EDL933ΔZ5898. The radius of the chemotactic ring of the complemented strain (EDL933ΔZ5898+ pBADZ5898) was 1.70 cm (Figure 4 B). The decrease of motility was almost complemented back by the plasmid pBADZ5898 expressing Z5898. The decrease between wild type and Z5898 deletion mutant was significant (P<0.01). This difference was not due to the growth rate which was similar among the mutant, the complemented strain and the wild type.

To investigate whether the reduced motility of the EDL933ΔZ5898 mutant was due to a decrease of surface flagella, bacteria were inspected by TEM to visualize surface flagella. while most EDL933ΔZ5898 mutant showed no flagella on the cell surface (Figure 4C). At a magnification of 9,700 times, 30 fields of view were randomly selected and about 50–200 cells counted, 90% Z5898 deletion mutant were found to have no flagella. The remaining 10% of the cells had 1 to 2 flagella. In contrast most of the cells from the wild-type EDL933 had surface flagella although the number of surface flagella was limited to between one and three. The electron microscope results suggest that the biosynthesis of flagella diminished in the Z5898 deletion mutant under the culture condition used in this study.

However, the surface flagella expression might differ from the *fliC* expression at transcriptional level as the surface flagella represent translational and post translational expression. Therefore the expression of *fliC* was examined in the wild type, mutant and complemented strain by immunoblotting (Figure 4D). The results showed a decrease of FliC protein in Z5898 mutant which was complemented successfully. These results are consistent with the swimming motility data and confirmed that Z5898 is involved in the upregulation of flagellar synthesis.

### Z5898 affects the expression of fliC but not its upstream regulator fliA

**Figure 5:**
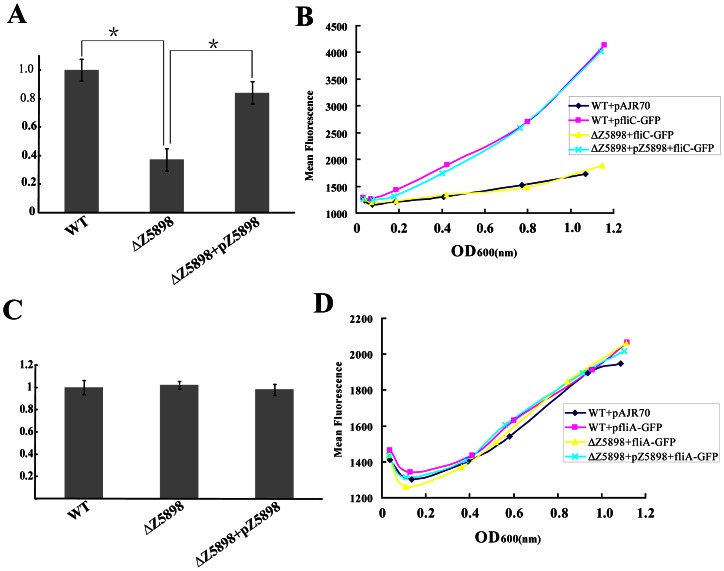
Transcriptional studies of *fliC* and *fliA*. (A) Expression of the *fliC* gene assessed at the mRNA level by quantitative reverse transcription PCR. Relative mRNA expression of *fliC* was normalized to that of the housekeeping gene *gapA*. Results represent mean values standard deviations (SD) for three independent experiments. Differences were analyzed for significance using T-test with significant difference between two strains (P<0.01) indicated by a * with a linked line. (B) Measurements of the *fliC* promoter activity in EDL933, isogenic Z5898 deletion mutant and complemented strain. A 340 bp *fliC* promoter fragment was cloned into the promoter-less green fluorescence protein (GFP) plasmid pAJR70 to create transcriptional fusion plasmid pAJRfliC. The fluorescence produced by each strain and corresponding OD_600_ was measured every 60 minutes. Fluorescence data were plotted against the mean OD_600_ measurement. The promoter-less plasmid pAJR70 was used as control. WT, wild-type EDL933; ΔZ5898, Z5898 deletion mutant. (C) Expression of the *fliA* gene by qRT-PCR. Data was also normalized using *gapA* expression as in A. Error bar shows the standard deviation from three independent experiments. (D) Measurements of the *fliA* promoter activity in EDL933, isogenic Z5898 deletion mutant and complemented strain. A 464 bp *fliA* promoter fragment was cloned into the promoter-less green fluorescence protein (GFP) plasmid pAJR70 to create transcriptional fusion plasmid pAJRfliA.

To investigate how Z5898 affects flagella synthesis, *fliC* transcription in EDL933 and EDL933ΔZ5898 was examined by qRT-PCR. The relative expression of *fliC* was normalized to that of a housekeeping gene, *gapA*. The qRT-PCR results showed that *fliC* mRNA expression in EDL933ΔZ5898 was down-regulated significantly in the mutant (Figure 5A), consistent with the proteomic data (Figure 3). The level of *fliC* transcription was complemented back by pBADZ5898 (Figure 5A). To test whether the *fliC* promoter activity was affected by Z5898, a plasmid expressing the *fliC* promoter – GFP fusion (pAJRfliC) was constructed and transformed into the wild type EDL933, Z5898 deletion mutant (EDL933ΔZ5898) and the Z5898 complemented mutant strain (EDL933ΔZ5898+pBADZ5898). The GFP fluorescence was measured over a 5 hour growth period (Figue 5B). The *fliC* promoter was repressed in EDL933ΔZ5898 but complemented back by pAJRfliC. Note that a small increase of fluorescence was seen in the negative control and the mutant due to cell growth. The *fliC* promoter – GFP fusion results and the qRT-PCR results suggest that Z5898 affects *fliC* expression at transcription level through modulation of the *fliC* promoter activity.

We further examined the upper level control of the *fliC* expression, the regulatory Class II gene, *fliA*. Transcriptional expression of *fliA* was analyzed by measuring *fliA* transcripts and *fliA* promoter activity. *fliA* transcripts among the wild-type, EDL933ΔZ5898 and the complemented strain were compared using qRT-PCR. There was no significant difference between wild-type and EDL933ΔZ5898 in transcriptional levels (t test, *P*>0.05, Figure 5C). A fluorescence plasmid containing the *fliA* promoter with GFP fusion was constructed and designated as pAJRfliA and introduced into the respective strains, the wild-type (EDL933), the deletion mutant (EDL933ΔZ5898), the complemented strain (EDL933ΔZ5898+pBADZ5898). The GFP fluorescence was measured over a 6 hour period, an overall increase in fluorescence was observed due to cell growth as in the case with the *fliC* promoter experiments, but the *fliA* promoter activity showed no significant difference between the wild-type and EDL933ΔZ5898, and between EDL933ΔZ5898 and the complemented strain (Figure 5D).

## Discussion

The *E. coli* O157:H7 genome contains many O islands with a few known to be important for virulence [1]. However, many OIs are of unknown functions and little is known of their evolutionary histories. In this study, our comparative genome analysis showed that the OI-172 is a composite island with progressive gain of genes since O157:H7 evolved from its ancestral O55:H7. A partial OI-172 was also found in 2 unrelated *E. coli* strains and 2 *Salmonella* strains. We further constructed a deletion mutant to study the function of the OI-172 targeting Z5898 which encodes a putative DEAH box RNA helicase. The Z5898 deletion mutant had reduced flagellin expression as revealed by 2-D gel electrophoresis of whole cell proteins. This repression effect on flagella was complemented back by pZ5898 expressing the Z5898 gene. Z5898 regulates the flagella based motility by exerting its effect on *fliC* at transcriptional level as indicated by qRT-PCR and promoter activity by measuring GFP production. Therefore we conclude that the OI-172 is a motility associated O island and hereby name it MAO.

Z5898 was found to have a positive effect on *fliC* expression and upregulates motility in this study. Assembly of flagella is controlled by a complex regulatory circuit that involves three classes of temporally regulated gene products. Many flagellar regulators act on class I regulatory genes *flhDC* at transcriptional level such as MatA [23], CRP [24], [25], H-NS [25], HdfR [28], QseBC [30], DksA and ppGpp [32] and sRNAs [33], which then regulates class II regulatory gene, *fliA*, the upstream regulator of *fliC*. We examined the *fliA* gene expression by qRT-PCR to measure the transcripts and a *fliA* promoter – GFP fusion to measure the *fliA* promoter activity. No difference was observed in either the amount of *fliA* transcripts or the *fliA* promoter activity. This indicated that the *fliA* promoter is not affected by the Z5898 gene. This result demonstrated that Z5898 regulates *fliC* rather than at class II regulatory level. It is likely that Z5898 is involved in processing *fliC* transcript to increase its expression, considering Z5898 is an RNA helicase. This inference was confirmed at *fliC* transcriptional level by qRT-PCR and GFP fusion plasmid pAJRfliC. It was reported that HrpA, a DEAH-box RNA helicase, was involved in *daa* mRNA cleaving and consequently upregulates the *daa* expression from the F1845 fimbrial operon [45]. Z5898 might also participate in *fliC* mRNA processing in *E. coli* O157:H7. However it is also possible that Z5898 acts indirectly affecting the *fliC* expression by interacting with other regulators such as H-NS and Z0021 which is located on OI-1.

Beyond the classic regulatory mechanisms of flagellar synthesis [18], there seems to be a far more intricate regulation of flagellar synthesis in O157:H7. A recent report revealed that Z0021 encoded by OI-1 of O157:H7 also plays a role in regulating flagellar synthesis [13]. The repression effect of Z0021 on motility can be restored by *flhDC* [13], suggesting that Z0021 functions at class II regulatory level in interaction with the FlhDC regulatory complex. In contrast, Z5898 encoded by the MAO island seems to activate the expression of flagella by up-regulating flagellar synthesis. Thus Z5898 and Z0021 exert an interesting antagonistic effect on motility and their potential interaction in controlling motility function will be investigated in future studies. Interestingly, this study also highlights the potential roles of the O islands in modulating O157:H7 flagellar synthesis and possibly virulence.

Bacterial flagella provide swimming and swarming motilities and also play a central role in adhesion, biofilm formation, and host invasion [46]. The flagellum of *E. coli* O157:H7 is known to playing a role in enhancing shedding of *E. coli* O157:H7 in a 1-day-old specific-pathogen-free chicken model [47]. *E. coli* O157:H7 flagellum acts as an adhesin to intestinal epithelium and involves in crucial initiating step for colonization [48]. The H7 flagellin has a role in provoking mucosal inflammation. It activates MAP kinase signalling pathways and the transcription factor NF-kB, leading to secretion of proinflammatory chemokines by human intestinal epithelial cells [49], [50]. Thus additional control of the flagellar synthesis instigated by Z0021 and Z5898 encoded on the O157:H7 specific OI-1 and OI-172 which represses and activates flagella expression respectively may enhance the role of flagella in the pathogenesis of O157:H7. Timing of the flagella expression may affect virulence. Turning it on during early stages of infection may aid colonisation while turning it off helps immune evasion once infection is established. In *Salmonella*, a recent study showed that an overexpression of either FliC or FlhDC alone, and co-expression of the two, significantly attenuates *Salmonella* virulence [51]. However further studies are required to determine the precise mechanisms and the timing of the control by both Z0021 and Z5898 and their regulatory roles in flagella synthesis *in vivo*.

The role of DEAH box RNA helicase in virulence and adaptation has hardly been studied. It is quite likely that Z5898 regulates other genes in addition to controlling flagellar synthesis. Proteomic comparison of the Z5898 deletion mutant and wild-type EDL933 revealed that other proteins may be differentially expressed and thus Z5898 is likely to have a wider role in the regulation of gene expression in O157:H7 and will be investigated in future studies. A recent report revealed that HrpA, a DEAH-Box RNA helicase, in the lyme disease spirochete *Borrelia burgdorferi* is involved in regulation of over nearly 200 genes [52]. Deletion of the *hrpA* gene renders *B. burgdorferi* avirulent in experimental mouse infections, further highlighting the role of DEAH-Box RNA helicase in virulence [52].

There are possibly other important virulence factors encoded by the MAO island. Using signature-tagged transposon mutagenesis, Dziva *et al* reported that the Z5886 transposon mutant failed to survive *in vivo* passage through calves suggesting that Z5886 plays a role in colonization [53]. It is interesting to note that Z5886 is not in O55:H7 which is rarely found in cattle. Thus the results obtained by Dziva *et al* and ours suggested that the MAO island is a putative pathogenicity island and may play a role in adaptation to the life style in the cattle intestinal tract.

In conclusion, we found that the OI-172 (MAO island) is a motility associated island and MAO island encoded Z5898 up-regulates the flagella based motility by exerting its effect through *fliC*. The MAO island is a composite island with progressive gain of genes since O157:H7 evolved from its ancestral O55:H7.
